# Neutron Crystallography for the Study of Hydrogen Bonds in Macromolecules

**DOI:** 10.3390/molecules22040596

**Published:** 2017-04-07

**Authors:** Esko Oksanen, Julian C.-H. Chen, Suzanne Zoë Fisher

**Affiliations:** 1 Science Directorate, European Spallation Source ERIC, Tunavägen 24, 22100 Lund, Sweden; esko.oksanen@esss.se; 2 Department of Biochemistry and Structural Biology, Lund University, Sölvegatan 39, 22362 Lund, Sweden; 3 Bioscience Division, Los Alamos National Laboratory, Los Alamos, NM 87545, USA; chen_j@lanl.gov; 4 Department of Biology, Lund University, Sölvegatan 35, 22362 Lund, Sweden

**Keywords:** hydrogen bond, neutron crystallography, structural enzymology, nuclear density maps, solvent networks

## Abstract

The hydrogen bond (H bond) is one of the most important interactions that form the foundation of secondary and tertiary protein structure. Beyond holding protein structures together, H bonds are also intimately involved in solvent coordination, ligand binding, and enzyme catalysis. The H bond by definition involves the light atom, H, and it is very difficult to study directly, especially with X-ray crystallographic techniques, due to the poor scattering power of H atoms. Neutron protein crystallography provides a powerful, complementary tool that can give unambiguous information to structural biologists on solvent organization and coordination, the electrostatics of ligand binding, the protonation states of amino acid side chains and catalytic water species. The method is complementary to X-ray crystallography and the dynamic data obtainable with NMR spectroscopy. Also, as it gives explicit H atom positions, it can be very valuable to computational chemistry where exact knowledge of protonation and solvent orientation can make a large difference in modeling. This article gives general information about neutron crystallography and shows specific examples of how the method has contributed to structural biology, structure-based drug design; and the understanding of fundamental questions of reaction mechanisms.

## 1. Hydrogen Bonds in Protein Structures

A hydrogen bond (H bond) is established through the sharing of an H atom that is covalently attached to an electronegative donor (D), with a different electronegative acceptor atom (A), and can be written as D-H…A. H bonds are one of the major drivers that participate in the correct folding and organization of secondary and tertiary structural elements in proteins. In addition to their fundamental role in structure, H bonds are also intimately involved in protein:ligand binding interactions and also form the basis for most types of enzyme-catalyzed reactions [1]. Proteins contain many different kinds of H bond donors and acceptors, from the backbone amide and carbonyl groups, solvent, to the side chains of most amino acids residues. H bonds are directional, which is the driving force behind the specificity of molecular recognition processes in ligand binding [2].

Studies in the 90s aimed at investigating the H bond frequency between H bond donors/acceptors and have been carried out on a representative set of protein crystal structures available at the time. It was observed that most donors and acceptors engage in H bonds, with only a small percentage remaining as unsatisfied atoms. Many amino acid side chains can and do make multiple H bonds, e.g., Lys, Arg, Asp, while others do not. For protein structure refinement and prediction calculations this is an important consideration to include [3].

From a theoretical chemistry point of view, the H bond interaction energy can essentially be described as being composed of a combination of covalent and weak interactions, e.g., van der Waals forces [2,4]. The energy associated with an H bond is 2–10 kcal/mol. While this may seem insignificant it is important to remember that these bonds are flexible and dynamic in that they are constantly broken and reformed under a range of temperatures and physiological conditions [2]. There is quite a large variation in H bond properties as observed in protein structures, especially in bond angles when comparing secondary structure elements to side chain characteristics. The D…A distances are typically between 1.7 and 2.4 Å, and the angle at the H atom can be anywhere from 130° to 170°, compared to ~150° at the acceptor [5]. As H bonds are difficult to directly observe, they are generally inferred when the donor-acceptor distance is less than ~3.5 Å, and the angles at the donor/acceptor are >90° [1]. Simple H atom sharing between two electronegative atoms represents the most common type of H bond, however, it is now accepted that there are additional types of weaker H bonds; e.g., C-H groups as donors, N-H…S, and C-H…π interactions with aromatic amino acid residues as acceptors [1,6]. A study conducted using NMR spectroscopy, DFT calculation, and data mining of high resolution protein crystal structures revealed that C-H…π type interactions are in fact quite common in proteins. These interactions are H bond-like in nature and occur between the methyl groups of Ile, Leu, and Val and the delocalized π electrons in aromatic residues. The results showed an average of four of these for every one hundred residues and are a functionally important part of protein structures [7]. H bonds in macromolecules are challenging to observe and classify, accordingly the criteria for identifying them in proteins are quite broad, as explained above. Another type of H bond that is commonly found in protein structures is the bifurcated H bond. In this case the slightly positively charged H atom is delocalized between two acceptor atoms. These are most often seen between Ser/Thr side chains with Asp/Glu as acceptors [8]. This indicates that H bonds cannot be identified only based on geometric criteria and that some additional structural or chemical knowledge is needed to fully understand all the H bonds in a protein. In recent years, bifurcated H bonds have also been directly observed in neutron protein crystal structures between Tyr and solvent, and Lys and Thr [9,10]. 

H atoms are abundant in macromolecules, comprising roughly half of the elements that proteins are composed of (C, H, N, O, S). However, despite their abundance and structural-functional importance, including but not limited to H bonding, electrostatic interactions, solvent coordination and solvent activation, H atoms and their interactions are very difficult to directly observe. This is especially true for X-ray crystallographic studies where information regarding H bonds are usually derived based on their heavy atom neighbor positions and the distances between them. The direct determination of H atom positions in protein crystal structures is restricted mainly due to their low X-ray scattering power, their inherent mobile nature, and limited crystallographic resolution [11].

## 2. Neutron Crystallography and Hydrogen Atoms

Neutron protein crystallography (NPX) is well-suited to the determination of the three-dimensional structures of proteins due to their sensitivity to the light atom H and lack of radiation damage at room temperature. NPX is conceptually very similar to standard X-ray crystallography, and crystallographic resolution is understood in the same way. The main difference between the methods lies in how neutrons are scattered from atoms. X-rays are scattered from the clouds of electrons around atomic nuclei, and the magnitude depends on the atomic Z number leading to heavy atoms scattering X-rays better than light atoms (Table 1). In practice this most often means that H atoms are invisible, except for ultra-high resolution structures that are exceptionally well-ordered, i.e., display low crystallographic B factors. Neutrons are scattered from atomic nuclei and the magnitude is independent of the Z number. Table 1 shows neutron scattering lengths for the most common atom types found in proteins. Coherent scattering lengths of neutrons are very similar in magnitude for C, N, ^1^H (and ^2^H or D), and O, making it as easy to observe C as H (or D) [11,12].

In practice this means that even at medium (2.0–2.5 Å crystallographic resolution) it is routine to readily observe the three dimensional positions of H atoms. By extension this means that neutrons are also used to study H bonds [14]. The isotope of ^1^H, deuterium (^2^H or D), has superior neutron scattering characteristics, making it optimal to replace H with D whenever possible (Table 1). The presence of ^1^H presents several practical problems for crystallographic data collection. The large incoherent cross section (Table 1) contributes to background as the incoherently scattered neutrons do not contribute to measured Bragg reflections. In addition, the coherent scattering length is negative and leads to troughs instead of peaks and can produce cancellation of positive peaks of adjacent atoms, complicating and confusing analysis of nuclear density maps when ^1^H is present. These effects can be minimized or even eliminated by D exchange (partial deuteration) or expression of the target protein under fully deuterated conditions (perdeuteration). Deuteration in general leads to higher quality maps and enables less ambiguous data analysis and structure interpretation [11,13].

Out of all H atoms in a protein, ~25% are titratable and can be exchanged by vapor H/D exchange or by soaking crystals in D_2_O-containing solutions. These include H found in the bulk solvent, ordered solvent, polar and charged amino acid side chains, and the protein backbone. Neutron structures derived from H/D exchanged proteins represent the vast majority of deposited structures in the PDB [11]. The remaining 75% of H atoms are non-exchangeable, typically found attached to carbons and have to be incorporated as D during protein expression. This is routinely done by growing *E. coli* cultures for protein expression in deuterated minimal media, such as M9, composed of stock solutions and components dissolved in D_2_O [11]. For the highest level D incorporation (~99%) it is best to use a perdeuterated carbon source, e.g., perdeuterated glycerol or glucose. This is very costly however and ~85% incorporation can be achieved by using deuterated M9 minimal media but supplementing with an unlabeled carbon source [15]. 

Cultures can be adapted in progressively increasing amounts of D_2_O or grown to high densities and switched to deuterated media just prior to induction of protein expression. All proteins are then extracted and purified as usual, under hydrogenous conditions, with lost D atoms back-exchanged at a later point. Depending on the strategy chosen the resulting protein will have varying degrees and distribution of D incorporation. While perdeuteration is preferable from a theoretical point of view, there are practical drawbacks that make it difficult to achieve in practice and the low numbers of neutron structures determined from perdeuterated proteins reflects this. Although perdeuterated proteins are nearly identical in structure to their hydrogenated counterparts, the proteins are often found to have altered chemical properties, such as reduced solubility and stability. Deuterated cultures also grow slowly, and perdeuterated protein yields are generally lower. Perdeuteration also introduces the need to fine-screen or rescreen crystallization conditions under perdeuterated conditions [16].

As there is no radiation damage with neutrons used for protein crystallography applications, it is routine to collect data at room temperature. This feature makes sample preparation for data collection simple as crystals can be mounted in quartz capillaries and subjected to H/D exchange in the capillary prior to exposure to neutrons. To achieve this, crystals are mounted in capillaries and a liquid plug of deuterated mother liquor is injected on either side of the crystal prior to sealing the capillary. The pitfalls of finding protective cryogenic solutions and the actual freezing of crystals are also avoided [11,16].

**Figure 1:**
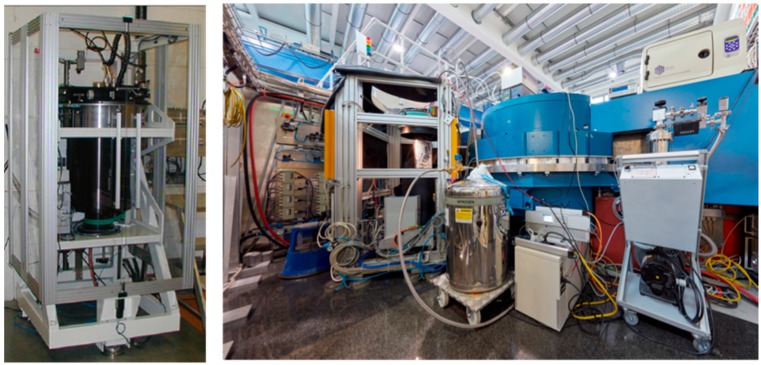
Neutron protein crystallography instrumentation. (**Left**) photograph of the LADI-III Quasi-Laue diffractometer, located at the Institut Laue-Langevin, Grenoble FR [19]; (**Right**) photograph of the BIODIFF monochromatic diffractometer, located at Heinz Maier-Leibnitz Zentrum, Munich DE. Re-used with permission from the photographer, Bernhard Ludewig.

Globally there are only a handful of instruments available that are specifically optimized for macromolecular crystallography. In the USA there are currently two instruments, both located at Oak Ridge National Laboratory (ORNL, Oak Ridge, TN, USA). The IMAGINE instrument is located at the HFIR reactor source, while MaNDi is found at the Spallation Neutron Source. In Japan there is the iBIX instrument at the Japan Proton Accelerator Research Complex (J-PARC, Tokai, Japan). In Europe there are currently two instruments operating at reactor neutron sources (Figure 1). Depending on the source and how the instrument is set up, data can be collected with monochromated (single wavelength) method or with a white Laue (multi-wavelength) method. The LADI-III (ILL, Grenoble, France) instrument uses a quasi-Laue spectrum, while BIODIFF (MLZ, Munich, Germany) uses a monochromated beam. However data is collected, the resulting neutron diffraction data sets from different instruments are remarkably comparable and can be refined using standard crystallographic software packages. After careful crystallographic refinement and analysis one can readily observe the three-dimensional structural details of H/D atoms, H bonds, water molecules and their interactions, the charged state of amino acids residues and their interactions, and the details of ligand binding. All this information is essential for the study of enzyme mechanism and drug binding, making neutron protein crystallography a powerful tool in structural biology [17,18].

In general the technique is still underutilized, with only ~100 PDB entries resulting from neutron diffraction studies. This is largely due to three major bottlenecks: (1) neutron scattering is a flux limited technique and accordingly requires long data collections times, ~5–20 days depending on the instrument; (2) quite large crystal volumes are required for most instruments (>0.5 mm^3^ on average); and (3) there are only a handful of instruments available worldwide to external users. Advances in sources, beamlines, and detector technologies are easing the way forward with much shorter data collection times from smaller crystals [11,16,17,18].

This review presents a number of topical examples of different kinds of structural and functional information that can be obtained with neutron crystallography, specifically how they relate to the study and importance of H bonds. Examples included are meant to span a broad area in structural biology and include systems where ordered solvent and H bonded solvent network are intimately linked to catalysis. Fluorescent and chromoproteins are also shown as excellent cases where optical properties are solely determined by changes in H bonding around a chromophore. Several enzymology examples are discussed in a later section, highlighting the importance of knowledge of H bonds and being able to determine how they change during catalysis. Due to the strong scattering of D, neutrons have also been used to “see” exotic solvent species such as the elusive hydronium ion (H_3_O^+^ or D_3_O^+^) and protons (H^+^ or D^+^ containing no electrons and therefore invisible with X-rays), an important proposed partner in certain kinds of enzyme-catalyzed reactions. Concluding remarks offer a forward-looking perspective on the place of neutrons in current-day structure biology.

## 3. Solvent Networks and H Bonding to Proteins

### 3.1. Ultra High Resolution Neutron Crystallography

**Figure 2:**
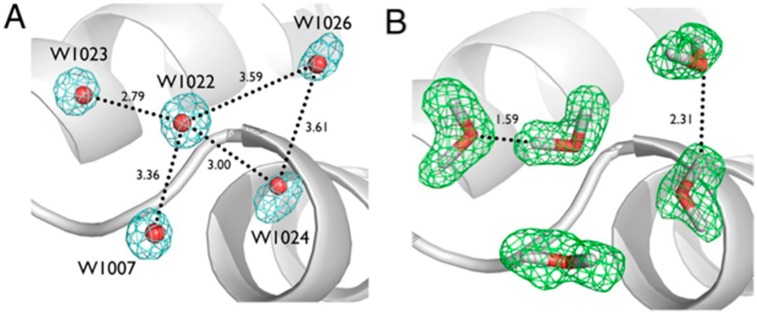
Ultra-high resolution X-ray and neutron crystallographic studies of H/D exchanged crambin, showing (**A**) details of possible H bonds between five ordered water molecules in crambin as observed in the X-ray crystal structure; and (**B**) observed H bonds between water molecules as seen in the neutron crystal structure. Figure reproduced from [20].

Ultra-high resolution (1.1 Å) neutron diffraction data was collected on a large crystal of the small protein crambin (46 residues, 4.7 kDa), isolated from seeds of *Crambe abyssinica*. The crystal had been exchanged against fully deuterated mother liquor prior to data collection. The very high resolution of the data allowed for a nearly complete inventory (~95%) of all H and D atoms in the protein [20]. Having unambiguous structural positions for the light atoms also allowed for easy interpretation and orientation of solvent molecules, with the ability to directly observe H bonding networks (Figure 2).

Even with the highest resolution X-ray structures, it remains difficult to visualize H atoms due to their low Z and proximity to heavier atoms, and as such, it is difficult to accurately model H bonding networks. In the case of crambin there is a 0.48 Å resolution crystal structure of crambin available in the PDB (3NIR), and also the recently collected 0.38 Å X-ray diffraction data set [21,22]. Even in these extraordinary cases it was still only possible to observe around two-thirds of all H atoms in the F_o_ − F_c_ difference maps. Inferring H atom positions and the H bonds they may be involved with can be misleading, as exemplified in the study of crambin (Figure 2A,B). There are five ordered water molecules seen in ultra-high resolution X-ray crystal structures of crambin with very well-defined oxygen atom positions but no definitive information on location of the H (or D) atoms. The dashed lines in Figure 2A indicate putative H bonds between these waters. Further inspection of the nuclear density maps showed that there are in fact only two H bonds between these waters (Figure 2B), and not five as can be interpreted based on the X-ray data alone. By taking advantage of the high resolution of the data, together with the strong neutron scattering exhibited by D atoms, it was possible to model the anisotropic vibrational behavior of a selected number of D atoms in crambin. Qualitatively, the D atoms were on average more anisotropic than their bonded, neighboring heavy atom [20].

### 3.2. H Bonded Water Networks in Proton Transfer

Human carbonic anhydrase II (HCA II) is one of 15 expressed isoforms found in humans. HCA II (261 residues, ~29 kDa) is a Zn-dependent enzyme that catalyzes the reversible reaction of carbon dioxide hydration to form bicarbonate and a proton. In general, HCAs are found throughout the human body, from blood to the brain, and are involved in many and diverse physiological reactions [23]. The HCA II active site has been studied extensively and defined by decades of biochemical and biophysical characterization, including X-ray crystallography, mutagenesis, NMR, and kinetics. HCA II is one of the fastest enzymes with a k_cat_ of 10^6^ and k_cat_/K_M_ approaching the diffusion limit. The reaction follows a ping-pong mechanism and is composed of two half reactions: carbon dioxide hydration followed by a proton transfer step. The rate-limiting proton transfer portion of the catalysis is mediated by a very well-ordered H-bonded water network that spans over ~8 Å and connects catalytic (Zn-coordinated) water to the bulk solvent via a proton shuttling residue, His64. The active site cavity is lined with several hydrophilic amino acid residues crucial for maintaining proper active site chemistry, geometry, and for coordinating the solvent network. The result is a well-tuned environment that allows fast and very efficient proton transfer [23].

**Figure 3:**
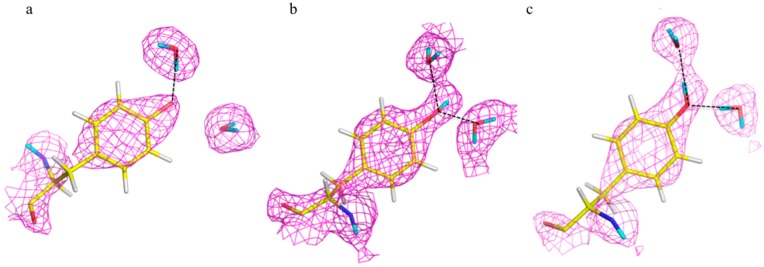
Three different states of Tyr7 in human carbonic anhydrase II. (**a**) Tyr7 is deprotonated at high pH; (**b**) Tyr7 is neutral at neutral pH but involved in a bifurcated H bond; (**c**) at pH 6.0 Tyr7 is still neutral and involved in a conventional H bond, participating as both acceptor and donor. 2F_o_ − F_c_ nuclear density maps are shown in magenta and are contoured at 1.3σ level. PDB IDs used (**a**) 3kkx, (**b**) 3tmj, and (**c**) 4y0j.

Neutron studies of HCA II at high pH (pH 10) showed the H bonding details of the ordered solvent network. Consistent with the decidedly non-physiological pH of the study, the organization and H bonding in the solvent network was such that it could not support proton transfer between catalytic water and the proton shuttling residue His64. In addition it was observed that the side chain of Tyr7, one of the residues that line the active site and coordinate the water network, was deprotonated (Figure 3a) [24]. This was surprising, even though the pH of the crystallization was over 10, as the theoretical p*K*_a_ of Tyr in solution is ~11. Tyr7 was the only deprotonated Tyr and all seven other Tyr residues were neutral, as expected. 

Further neutron structures were solved at pH 7.8 and 6.0 to further probe the solvent network and changes in protonation of Tyr7 (Figure 3b,c) [9,24]. The pH 7.8 neutron structure showed a rearrangement of the solvent network, with all the waters now connected by H bonds, and neutral His64 poised to accept a proton. This pH “switch” indicated the pathway that an excess H^+^ can use to leave the active site. At pH 7.8 Tyr7 was neutral and involved in a bifurcated H bond with two solvent molecules (Figure 3b). At pH 6.0 the water network was unchanged compared to the neutral form, but Tyr7 had changed how it H bonds to the solvent to a more conventional configuration (Figure 3c). Another key observation was that of the proton shuttling residue, His64. At pH 6.0 it was directly observed that His64 was charged (doubly protonated) and flipped away from the active site and involved with a π-stacking interaction with Trp5. Comparison of the low pH form with the high resolution X-ray crystal structure of HCA II in complex with substrate carbon dioxide, it was seen that the active sites appeared similar in solvent positions and amino acid residue orientations. This suggests that protonating the active site by lowering the pH provides a glimpse of how the active site is poised to accept substrate.

The first neutron structural studies were followed up with NMR titration studies of ^13^C-Tyr labeled HCA II as a function of pH. It was possible to assign chemical shifts for all eight Tyr residues and the data confirmed the observation of a perturbed p*K*_a_ for Tyr7. Consistent with the crystallographic data Tyr7 had a severely perturbed p*K*_a_ at ~7, compared to ~10–12 for the seven other Tyr residues. The combined results of the high pH (pH 10), neutral pH (pH 7.8) and low pH (pH 6) forms revealed important details about proton transfer, amino acid residues protonation and H bonding, and how ordered solvent networks can respond and adapt to changing conditions [9]. These structural features could never be observed with X-ray crystallography alone as the ~1 Å X-ray structure did not indicate any explicit H atom positions, even on well-ordered residues. This study used pH as a variable parameter to mimic events that could occur during catalysis. Analysis of these data suggests an alternate catalytic model for how HCA II works. With a p*K*_a_ in the physiological range Tyr7 may very well be part of catalysis under normal pH conditions.

### 3.3. Chromoprotein Dathail and H Bonding

Fluorescent proteins (FP) occur naturally and display different fluorescent characteristics and are widely used in biological research as probes and reporters. Protein engineering, mutagenesis, and directed evolution have dramatically increased the variety and spectral properties of FPs. Green fluorescent protein (GFP) has a β-barrel structure made of 11 β strands with the chromophore located in a central pocket. The chromophore is formed after expression by the cyclization of 3 amino acids to form *p*-hydroxybenzylidenimidazolinone. The pocket around the chromophore is lined with amino acid side chains and ordered water molecules; mutagenesis can dramatically affect the spectral properties of variants. Some of these chromoproteins can photoswitch between fluorescent and non-fluorescent states by simple illumination by a specific wavelength of light. From X-ray analysis it is thought that switching from the fluorescent state is related to *cis*-to-*trans* isomerization of the chromophore, with *cis* corresponding to the ground state (fluorescent). Due to the structural rigidity of FPs it has been suggested that H bonds and related chemical environment around the chromophore play a fundamental role [25].

Neutron, X-ray and spectroscopic studies of a newly engineered FP, Dathail, revealed some interesting and unusual photoswitching behavior. Dathail has very high temperature stability, is less prone to aggregation, and readily crystallizes. Even more interesting is that the crystals tolerate photoswitching, allowing data to be collected of the “dark” (ground) and “light’ (metastable) states. Dathail is negative switching, with highest fluorescence in the ground state that is turned off when exposed to 497 nm light. Fluorescence emission spectra also showed these two states. The protein returns to the dark state through a very slow thermal relaxation process which takes several hours at 10 °C [26]. X-ray crystal structures of the dark state showed that the chromophore is very well ordered in the *cis* conformation. There was a dramatic difference in the light state where the chromophore, surrounding amino acid residues, and solvent were very mobile and disordered in this form.

X-ray structures alone did not provide insights into the photoswitching behavior of Dathail. The structure of the Dathail in the ground state is remarkably similar to those of other FPs, especially with regards to the chromophore region. The observed structural configuration is associated with fluorescence in other chromoproteins, however, Dathail is non-fluorescent in this form. As the differences seem to lie in H bonding patterns, neutron crystallographic data were collected from a large 10 mm^3^ H/D exchanged crystal of the dark (ground) state [26]. The fact that the ground-state chromophore of Dathail is clearly protonated and stable with the chromophore in the *cis* conformation challenges the consensus view that a stable, co-planar chromophore in the *cis* isomer leads to stable fluorescence, given that Dathail is essentially non-fluorescent. 

Neutron studies showed that the chromophore is protonated in the ground state and showed further details of water molecules, H bonds, and protonation states of certain residues in the chromophore pocket. Furthermore, there is an H bonded water network from the chromophore to the bulk solvent that may be involved with the transfer of excited state protons out of the pocket during photo-activation and helps to explain the lack of fluorescence in the ground state.

**Figure 4:**
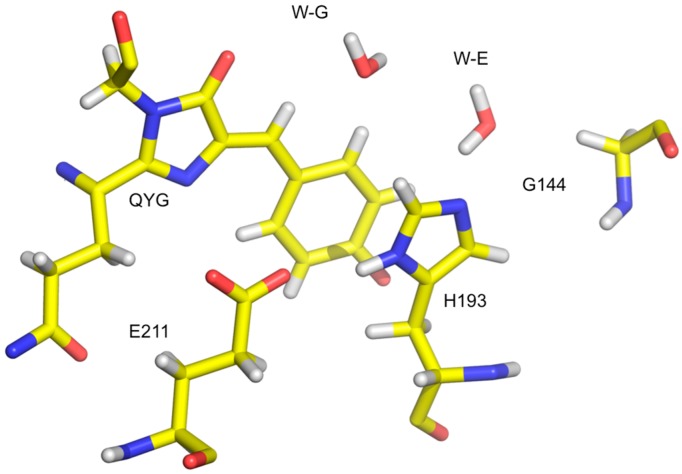
The chromophore pocket of Dathail shows interactions to amino acid residues and organization and orientation of key water molecules. Figure generated from PDB 4ebj [26].

The precise details of the water network around the chromophore are unique to Dathail. This is due to the presence of a Ser to Pro mutation at position 142. The waters mediate the de- and re-protonation of the chromophore. In other FPs the chromophores are thought to H bond to the Ser (or Thr) residue in this position, making it difficult to protonate. From these neutron studies it was also seen that His193 is neutral in the ground state. His193 is an H bond acceptor from a water molecule in the network, and is in turn a donor to Glu211. A Gly at position 144 affects how His193 is coordinated, especially compared to other chromoproteins where there is a Glu at this position. The imidazole ring of His193 is π-stacked with the chromophore itself, similar to other chromoproteins (Figure 4). However, the loss of a H bond due to a Glu to Gly mutation makes His193 more dynamic and less able to order the chromophore. In this study neutrons provided valuable insights into the chromophore pocket organization, the role of solvent molecules, and helped identify key H bonding interactions that are at the heart of this unique photoswitching behavior [26].

## 4. Unusual H Bonds and Solvent Species as Observed in Neutron Crystal Structures

### 4.1. Photoactive Yellow Protein: Short H Bonds vs. Low-Barrier H Bonds

Photoactive yellow protein (PYP) is a soluble 14 kDa protein from *Halorhodospira halophila* that absorbs light via its para-coumaric acid (pCA) chromophore. The absorption spectra for PYP is equivalent to the negative photo-dependent response of the organism, implicating PYP in its biological behavior. PYP has a reversible photocycle during which pCA undergoes *trans*-to-*cis* isomerization, causing remodeling of the H bonded network and proton transfer processes. Isomerization of pCA culminates in large structural changes of N-terminal regions of the protein. This means that H bonding is essential for photocycle kinetics in the protein. In the ground state pCA is stabilized by two H bonds between the pCA phenolate oxygen atom and residues Tyr42 and Glu46. High-resolution X-ray structural studies revealed these bonds to be rather short at 2.49 Å and 2.58 Å, respectively. It was suggested that these bonds represent short H bonds (SHB) [27].

As the H bonds become shorter there is a lengthening of the donor O-H covalent bond. The barrier decreases as the disorder over two protonation sites collapse to a centered single-well potential, the so-called low-barrier H bond (LBHB). In an LBHB the proton is shared between donor and acceptor atoms, making it behave like a covalent bond [28].

**Figure 5:**
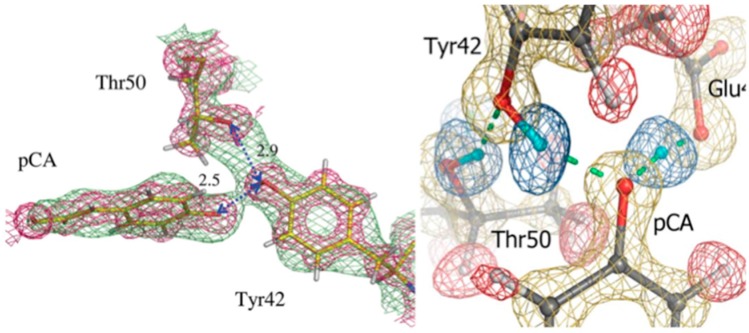
Nuclear density maps around the pCA of PYP. (**Left**) H bonds around the pCA in PYP as observed in the 2.5 Å structure [29]. Reproduced with permission from the International Union of Crystallography; (**Right**) High resolution neutron studies to 1.5 Å enable accurate determination of H/D atom positions and assignment of LBHB in PYP. Figure reproduced from [28].

Neutron crystallography was used to study PYP in ground state in order to investigate these short H bonds and to elucidate H atom positions in the area of the hydrophobic core [28,29]. The first neutron crystal structure of PYP was determined to 2.5 Å resolution, from a ~0.8 mm^3^ crystal that was H/D exchanged. From this first structure it was possible to elucidate some of the details around the pCA pocket, with pCA being deprotonated and the phenolate oxygen accepting H bonds from Glu46 and Tyr42. Tyr42 in turn is H bonded to Arg52. The observed H bond lengths to pCA were consistent with the high-resolution X-ray work, remarkable given the moderate resolution (2.5 Å) of the neutron structure (Figure 5) [29].

In 2009 a much higher resolution neutron structure of PYP was reported, to 1.5 Å resolution. Now it was possible to fully investigate the short H bonds around pCA to see if they could correspond to LBHB [28]. From detailed investigations of omit F_o_ − F_c_ and 2F_o_ − F_c_ nuclear density maps it was found that the SHB between Glu46 and pCA could be a LBHB, while the Tyr42-pCA H bond was better classified as a short ionic H bond (SIHB).

It is thought that the deprotonated/charged pCA represents an isolated charge in the otherwise hydrophobic pocket of PYP with Arg52 acts as a counter-ion. However, in the high-resolution neutron structure this Arg was neutral. It was postulated that the SIHB and LBHB compensate for this and provide structural stability to PYP. LBHBs have been implicated in other systems for being part of catalysis and/or stabilization of intermediates. In this case the proposed LBHB may stabilize an isolated charge and participate in proton transfer during the photocycle of PYP [28]. Comparison of the two studies also shows, unsurprisingly, that higher resolution neutron crystallographic data allows for more accurate and detailed studies.

### 4.2. Unusual H Bonds as Seen with Neutrons

Recent neutron structures have revealed a wide variety of H bonding details, including LBHBs, SIHBs, and resonance-assisted H bonds. Despite over 40 years of structural, mechanistic, and computational work, there remains much to understand about this fundamental, yet varied interaction, found in protein structures and enzyme mechanisms. 

To study whether a LBHB was involved in catalysis, neutron studies were carried out on a serine protease, porcine pancreatic elastase (PPE), in complex with a peptidic inhibitor FR130180 [30]. The residues His57-Asp102-Ser195 compose the catalytic triad and are conserved in the serine proteases. In the first step of catalysis, the -OD1 of Ser195 makes a nucleophilic attack on a substrate carbonyl group. Next, a tetrahedral intermediate is formed through a covalent bond to Ser195 and the substrate carbonyl group. The tetrahedral intermediate is stabilized through electrostatic interactions that involve H bonds to backbone amides of Ser195 and Gly193. Together this forms a proposed oxyanion hole. Ser195 is activated for nucleophilic attack through a SSHB between side chains of His57 and Asp102 [31]. The tetrahedral intermediate is resolved through proton transfer from His57 to the leaving group, leaving an acyl-enzyme intermediate. The H bond between Ser195, His57, and Asp102 has been proposed to be a LBHB and has been investigated with high resolution X-ray crystallography and NMR spectroscopy, in different types of proteases such as trypsin and subtilisin. The studies were inconclusive and in some cases instead showed a SIHB where the short H bond (~2.6 Å) is observed. However, the shared H atom remains with the donor, and is not found equidistant between donor/acceptor as expected in a LBHB [32,33].

FR130180 binds to PPE with the same tetrahedral transition state intermediate as seen with substrate. The nuclear density maps show that His57 is protonated (charged) and involved in a short H bond with Asp102, however the shared D density is clearly located on -ND1 of His57, and is most likely a SIHB. In addition the neutron data showed the carboxyl oxygen (O32) of FR130180 is present as an oxygen anion (oxyanion) and was the first oxyanion in a tetrahedral intermediate observed at an oxyanion hole. At a substrate binding subsite, S4, a π-cation interaction is observed between Arg217 and the benzoyl ring of FR130180. Finally, there is an H bond between the –OH group of Thr175 and the carboxyl group of benzoic acid in the inhibitor. In this interaction, the carboxyl group is protonated and the O-D distance is longer than expected (1.21 vs. 1.02 Å), while being only 1.41 Å away from the -OD1 of Thr175. This configuration is consistent with another type of very strong H bond, the resonance assisted H bond (RAHB) [30].

**Figure 6:**
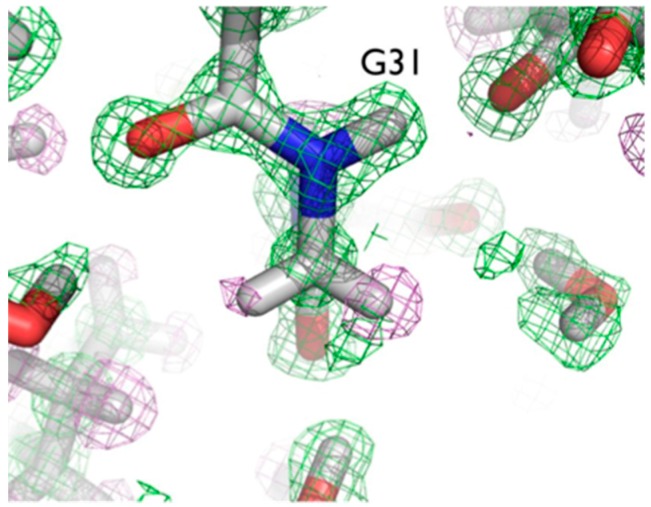
Nuclear 2F_o_ − F_c_ density for crambin residue Gly31, with negative (pink) density for H and positive (green) for other atoms, showing different degrees of H/D exchange for the two alpha carbons. Figure reproduced from [20].

The ultra-high resolution (1.1 Å) of the crambin neutron structure made it possible to clearly resolve H atom positions and the degree of exchange with D [20]. More accurately than in other neutron structures resolved to date, the relative occupancies of H/D at exchangeable positions could be refined, and solvent molecules and side chains could be identified and oriented. Of note were two particular regions of the protein with unexpected characteristics. In Gly31, the two Hs appear to have different degrees of exchange, based on the observed nuclear density for the two H atoms (Figure 6).

One interpretation of this is that there was partial exchange of the Cα-H against the deuterated solvent, leading to partial D occupancy, and the possibility of Cα-H…O H bonding. This can be attributed to differing chemical environments in the crystal, corroborated by solution state NMR studies.

The two Cα-H atoms in Gly31 were observed to have distinct NMR proton chemical shifts of 3.96 and 5.56 ppm in solution, reflecting different chemical environments for the two alpha hydrogens [34]. Since Gly does not have a side chain, they are relatively strong carbon acids (p*K*_a_ ~ 22), comparable to the side chain hydroxyl groups of Ser and Thr (p*K*_a_ ~ 18–20).

**Figure 7:**
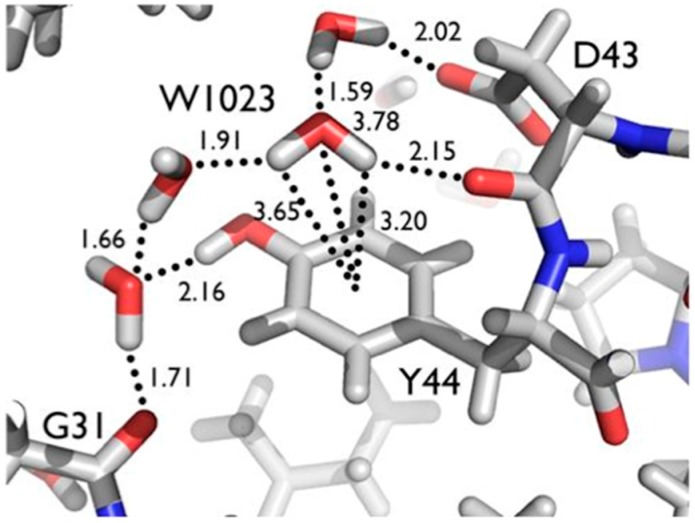
Crambin residue Tyr44 is making van der Waals contact with Ile33 on one side of the aromatic ring and is solvated on the other side. W1023 interacts with the π-system of residue Tyr44. D1 of W1023 is directly pointing Gly31 and different degrees of H/D exchange. Figure reproduced from [20].

Another series of potential Cα-H…O H bonds were observed in the β-strand region of crambin, e.g., between the carbonyl oxygen of Thr1 and the Cα-H of Ile34. These observations add to the evidence that these weak, but numerous, stabilizing H bond interactions serve to contribute to secondary and tertiary structure stability. Secondly, Tyr44 in crambin is a well-ordered surface residue that is solvated by a network of waters on the one side, and makes a van der Waals interaction with Ile33 on the other side. As shown in Figure 7, one of these ordered water molecules, W1023, has its D1 atom pointing towards the center of the aromatic ring, indicating a potential O-H…π H bonding interaction [20]. With improvements in X-ray and neutron instrumentation and crystallization approaches, ultra high-resolution crystallography will allow for H bonding interactions to be studied in greater detail and to better accuracy.

### 4.3. Protons (H^+^), Hydroxides (OH^−^), and Hydronium Ions (H_3_O^+^) in Proteins

Water (H_2_O), protons (H^+^), hydroxide (OH^−^) and hydronium ions (H_3_O^+^) are important players in many chemical and biochemical processes. They may be involved in a variety of different enzyme catalytic processes but are difficult, or in the case of H^+^, impossible to directly observe using X-rays. In electron density maps derived from X-ray crystallography, most of these hydrated proton forms are indistinguishable from each other as only the oxygen peak of these species are visible in electron density maps. Neutrons provide the appropriate tool to “see” the light atoms in solvent and make it possible to discern exotic solvent species from ordinary water.

Structure-function studies of xylose isomerase (XI) using neutrons made it possible to observe the first hydronium, hydroxyl, and proton ions in a biological system [35,36]. XI from *Streptomyces rubiginosus* is a 172 kDa homotetramer with 2 metal sites that bind divalent cations, usually magnesium, in the active site. XI catalyzes the interconversion of d-glucose to d-fructose, or d-xylose to d-xylulose, and is of considerable industrial interest for production of sweeteners and in the biofuel field [37]. Metal site M1 is coordinated by a number of waters, and Glu and Asp amino acid residues. Metal site M2 is coordinated by His, Asp, Glu side chains and a catalytic water molecule. During catalysis there are multiple steps: substrate binding and coordination adjacent to the two metal sites, sugar ring opening, movement of a H from sugar C2 to sugar C1 through a proposed hydride shift or donation of a hydride anion, and ring closure. The process culminates with the product leaving the active site. These steps necessarily involve complex changes in water and amino side chain positions, as well as movement of H atoms through protonation/deprotonation of water and active site residues [35,38].

Using a combination of different metal substitutions and by soaking in substrate or product molecules, it was possible to trap various intermediate stages that occur during catalysis by XI. In one such structure of the natural magnesium-containing enzyme in complex with the product xylulose, a hydroxyl anion was observed in place of the catalytic water at the M2 site. This observation supports the idea that the catalytic water molecule donated a proton during the isomerization step of catalysis and gave important clues as to the molecular events that occur upon isomerization [35].

Subsequent neutron studies looked at metal free XI at both pH 5.9 and 7.7 to help understand why the enzyme is inactive at low pH. XI is most active at ~pH 8, however for industrial applications it is desirable to engineer the enzyme to also be active at lower pH. It is known that at low pH the metals from M1 and M2 are expelled due to electrostatic changes in the area and this was also observed in the neutron crystal structures. Neutron studies were conducted to study how the active site looks in the absence of metal, and how the charged state of residues change as a function of pH.

**Figure 8:**
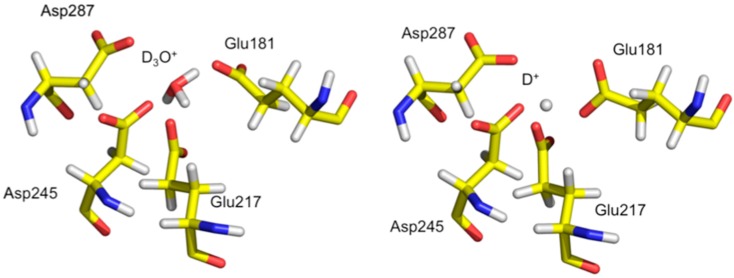
Atomic details of metal binding site M1 using neutrons at two different pHs. (**Left**) Coordination of hydronium ion D_3_O^+^ located in metal site M1 at pH 7.7 (from PDB ID 3kcj); (**Right**) Coordination of proton D^+^ located in metal site M1 at pH 5.9 (from PDB ID 3qza).

Analysis of the neutron structure at pH 7.7 showed that the metal at position M1 is replaced by a hydronium molecule (Figure 8) [36]. The hydronium ion templates the appropriate size and shape for metal binding at M1 and is coordinated by Glu181, Glu217, Asp245, and Asp287. In the pH 5.9 structure the hydronium ion has been dehydrated to leave a proton only in the M1 site. The proton is involved in a very short trifurcated H bond to coordinating residues and located closest to Glu217. At low pH the amino acid residues have collapsed around the site and does not have the proper geometry to accept an incoming metal ligand. Soaking in solutions of metal salts were also unsuccessful, providing further evidence that the low pH form of M1 is not able to bind metal, thus explaining the lack of activity of XI at low pH [37].

### 4.4. The Role of D_3_O^+^ Ions in Redox Processes

Rubredoxins are highly thermostable, small, mononuclear iron-sulfur cluster proteins found in both prokaryotes and eukaryotes. As redox proteins they serve as an important model system for the study of proton transfer in catalysis. Iron-sulfur proteins vary greatly in their redox potentials, reflecting differences in solvation, electrostatics, and H bonding. Neutron studies were initiated to obtain high resolution structures of both oxidized and reduced forms on rubredoxin, in order to study water molecules, H bonding, and to investigate the charged state of chemical groups in the protein as a function of oxidation state [39]. 

Inspection of the ~1.3 and 1.4 Å resolution nuclear density maps revealed several interesting structural features involved with protein stability in both oxidized and reduced forms of the protein. Four hydronium ions were observed in both oxidized and reduced rubredoxin structures. The first molecule, HYD1, was located near Leu51 amide group and involved with amide-imide tautomerization. The second one, HYD2, is found by Pro44-Pro19-Ser46. HYD2 is ~10 Å away from the iron-sulfur cluster and forms water-mediated H bonds to iron-sulfur coordinating Cys and adjacent Ser residues. In this way HYD2 is at the center of a tight H bonded group that connects water to the metal cluster coordinating residues. HYD3 also found near the iron-sulfur cluster (~9 Å), but near Pro25-Ser24. The last one, HYD4, is H bonded to Ala16 carbonyl group. Occupancy refinement of one of the D atoms indicates water/hydronium equilibrium. 

In summary then, three of the four hydronium ions are observed close to the protein main chain and another one forms the core of an intricate H bonded water network. One of these hydronium ions is located near main chain amide of Leu51 and is probably involved with redox driven tautomerization of amide to imide forms of the residue. In addition to the several hydronium ions seen in rubredoxin, there were two additional important protonation shifts observed, in residues Glu47, Asp13, and Asp15. Glu47 was seen to be unprotonated in the reduced form while being protonated in the oxidized form. This shifting D atom is also associated with HYD2. Asp13 on the other hand is unprotonated in the oxidized form while Asp15 is protonated. The observation of four hydronium ions and three shifting residues in close proximity to the iron-sulfur cluster suggest that these entities and surrounding solvent play important roles in charge transfer processes that occur in the change between oxidization and reduction in rubredoxin and associated redox processes [39].

With the growing use of neutron protein crystallography, it can be expected that more of these unusual solvent types will be directly observed and shed light on intricate and complex protein functions.

## 5. Structural Enzymology and H Bonds

Hydrogen atoms are central to enzyme mechanisms and catalysis often relies on the movement of H atoms, either through proton shuttling or hopping or through the breaking/making of H bonds. There are many examples of neutron crystallography helping to elucidate enzyme mechanistic questions and all cannot be covered here. In this section a few examples are discussed where using neutrons made a significant contribution with regards to hydrogen atoms and their interactions.

### 5.1. Cytochrome C Peroxidase

Heme peroxidases are widespread in nature and support a wide range of biological functions. They employ peroxide to oxidize a number of different substrates. Catalysis by heme peroxidases require the formation of two transient, highly oxidized ferryl (Fe(IV)) intermediates, the so-called Compounds **I** and **II**. These intermediates are applied widely in other oxygen-dependent catalytic heme-containing enzymes, including the cytochrome P450s, nitric oxide synthases, terminal oxidases and the heme dioxygenases [40]. It is important to understand the protonation state around the heme as this determines the reactivity and involvement of the enzyme.

Compound **I** and Compound **II** form sequentially during catalysis and represent different Fe-IV (ferryl) species. They are different in their oxidation state, specifically the porphyrin ring. It is thought that the species are Fe(IV)=O or Fe(IV)-OH and it has been challenging to characterize the identities definitively [40,41]. Photo-reduction of the iron has been observed in both Raman spectroscopy and X-ray crystallography, rendering the interpretation of the oxidation states derived from these methods unreliable. Neutron crystallography can provide the high resolution, unambiguous structural information required to characterize the iron oxidation state, without inducing photo-reduction in the samples [40,41]. To unravel the mechanism of the peroxidases, two related heme peroxidases have been studied with neutron crystallography. Cytochrome c peroxidase (Ccp) oxidizes the small protein cytochrome c by hydrogen peroxide, and the mechanistically and structurally related ascorbate peroxidase (APX), the oxidation of ascorbate [40,41].

Cytochrome c peroxidase is a well-studied model system for heme-containing oxidases, which play a key role in many metabolic processes. Various species along the reaction path can be characterized by spectroscopic methods and trapped by cryo-cooling for crystallographic structure determination. However, the protonation states—which are crucial for understanding the electronic structure—are not easy to infer even with the combination of spectroscopy and crystallography.

**Figure 9:**
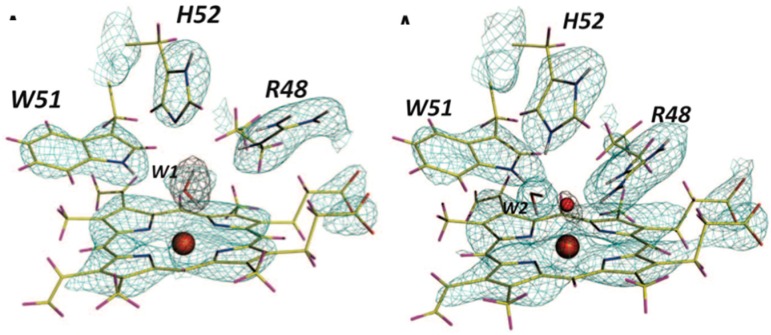
Neutron crystal structures of cytochrome c peroxidase in the ferric and Compound **I** form. (**Left**) nuclear density maps show that a heavy water, D_2_O, is bound to the heme; (**Right**) nuclear density maps show that oxygen is bound to the heme in the Compound **I** intermediate form. Figure reproduced from [41].

The structure from neutron diffraction study of the ferric state of the heme, determined at 2.4 Å resolution, showed a heavy water molecule (i.e., D_2_O) bound at the start of the reaction (Figure 9, left). Crystals were then treated with peroxide, known to induce formation of Compound **I**. The treated crystals were cryo-cooled to trap the intermediate and studied with neutrons. The neutron structure, determined to 2.5 Å resolution, clearly shows the oxygen to be unprotonated, consistent with the H bonding structure of the active site (Figure 9, right). The ferryl iron is then double bonded to an oxygen (Fe(IV)=O). The Trp 191 radical is protonated, so the radical species is a (protonated) π-cation radical [41].

Another recent neutron crystallographic study focused on determining the oxidation and coordination state of the Compound **II** intermediate in APX [40]. APX catalyzes the peroxide-dependent oxidation of ascorbate by using both Compound **I** and Compound **II** intermediates. APX and Ccp are highly conserved and Ccp has served as a model system for mechanistic studies in heme enzyme catalysis. The experimental challenges of studying Compound **II** are more easily overcome in APX compared to Ccp. In APX Compound **I** appears as a ferryl heme and a porphyrin π-cation radical and is readily distinguishable from Compound **II**, which contains only a ferryl species. This is different from Ccp where Compound **I** is a ferryl heme and a Trp radical. Careful X-ray analysis and microspectrophotometry, along with neutron crystallography, revealed the heme ligand to be hydroxyl. At the resolution limits of the crystallographic studies, it is not justified to assert that the refined distance (1.88 Å) corresponds to a hydroxyl bound to the heme. However, analysis of omit maps and lack of residual density led to the determination that Compound **II** in APX is indeed Fe(IV)-OH [40]. Together these results offer new insights on the mechanism of heme peroxidases, with implications for the entire family of enzymes. Importantly, for the first time, they offer unambiguous insights into the coordination and protonation state around the heme groups where other techniques have been unsuitable.

### 5.2. Urate Oxidase

Urate oxidase (EC 1.7.3.3) is an enzyme involved in purine metabolism. It is a co-factorless oxidase that catalyzes the reaction between uric acid and molecular oxygen to form 5-hydroxy-isourate (5-HIU), which in turn is hydrolyzed to allantoin.

**Figure 10:**
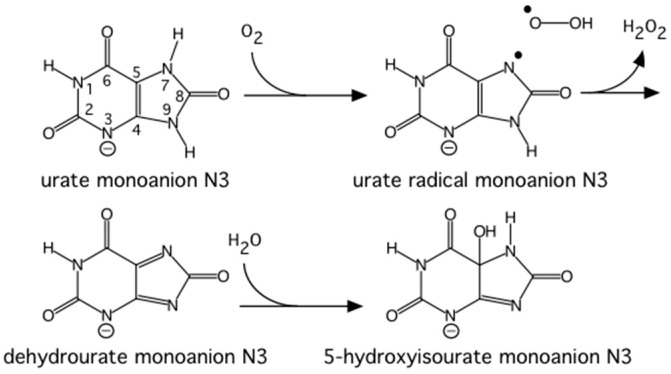
The reaction catalyzed by urate oxidase. Figure reproduced from [42].

**Figure 11:**
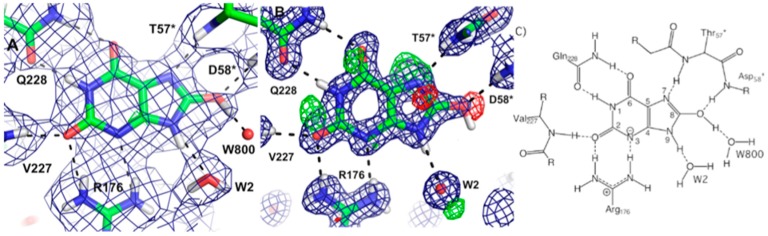
The 2.35 Å resolution 2mF_o_-DF_c_ nuclear density map of urate oxidase (**A**) compared with the 1.05 Å X-ray map (**B**); (**C**) a schematic representation of the surroundings of the urate substrate. Panels A and B reproduced from [42].

The reaction between oxygen and a urate dianion is spontaneous in solution, but the predominant protonation state of uric acid in solution is a monoanion with N3 deprotonated (Figure 10) [42]. Therefore the principal role of the enzyme is to deprotonate the urate substrate in proximity of the oxygen. Numerous X-ray structures were determined both with inhibitors that mimic urate as well as oxygen at atomic resolution. The results were difficult to reconcile with the biochemical data, since the site of the next deprotonation in solution, N7, is H bonded to a main chain amide and no feasible general bases can be found in the vicinity. The neutron structure at 2.35 Å resolution (Figure 11) showed that in fact the species in the active site was the enol tautomer of urate, 8-hydroxyxanthine.

It was shown by quantum chemical calculations that despite the enol tautomer being unstable in solution, the active site can stabilize it. Interestingly, the O-H was clearly pointing away from the purine plane, even though there was no H bonding partner observed to explain this behavior. There was however enough empty space for a water molecule, even though none were observed in the X-ray maps. Further quantum mechanics/molecular mechanics calculations were performed to understand this. It was not possible to obtain good geometry consistent with the high-resolution X-ray structure unless a water molecule was placed in proper H bonding geometry with respect to the O-H. This illustrates the synergies between crystallography and computational chemistry where computational methods can predict or fill in details when crystal structures give incomplete information. A water molecule was indeed observed in this position in an X-ray structure from anaerobically grown crystals [43].

**Figure 12:**
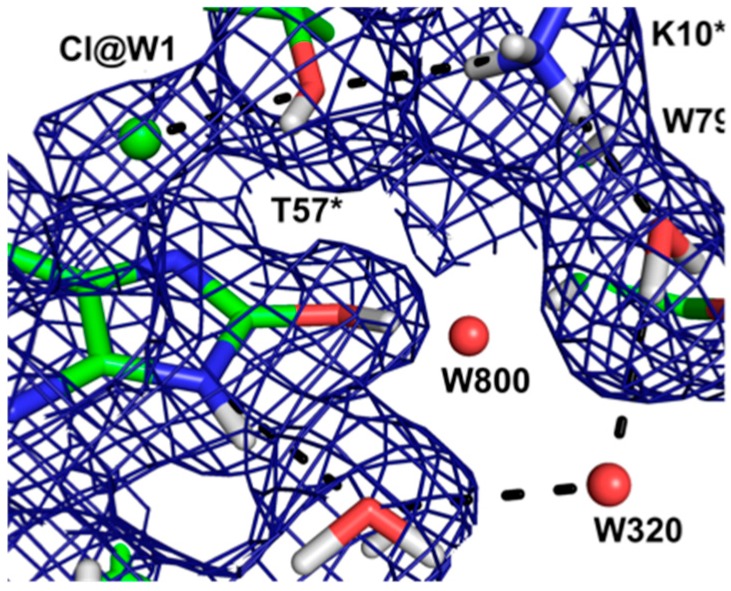
The postulated proton relay chain as seen in the 2mF_o_-DF_c_ nuclear density map. Figure reproduced from [42].

This result allowed a more plausible mechanism to be postulated where the deprotonation occurs on the oxygen instead of the nitrogen. One of the unsolved questions in the mechanism was always how an H atom from the N7 position can reach the oxygen site above the ring plane. A surprising observation in the neutron structure was that an H bonded network extended from N7 all the way to the oxygen site (that contains Cl^−^ in this structure), and the H atoms were disordered (Figure 12).

Nevertheless the X-ray structure showed that the oxygen atoms were well ordered. This is indicative of a proton relay chain that can easily pass the proton through three water molecules, a Lys and a Ser side chain. The existence of such a relay chain also explains how the oxygen site can be provided with a second proton to eventually form H_2_O_2_.

### 5.3. β-Lactamase

Bacteria have developed antibiotic resistance to β–lactam antibiotics by expressing an enzyme, β-lactamase, which breaks the amide bond within the lactam ring and renders the compound ineffective. This class of antibiotics inhibits cell wall synthesis by binding to bacterial penicillin-binding proteins (PBPs). The β-lactamases are divided into classes A through D. All but class B use a serine-reactive mechanism. The class A CTX-M extended-spectrum-β-lactamase (ESBL) has high hydrolytic activity against first, second, and third generation cephalosporins and monobactams. The enzymes work by using a general base mechanism to break the amide bond within the β-lactam ring [44]. One class A enzyme, CTX-M-44, was chosen for neutron studies to further investigate the role of active site Glu166, Ser70, and Lys73 in catalysis. CTX-M-44 is composed of 262 residues and has two conserved domains, with the active site located at the interface between the domains. It has been proposed that Glu166 acts as a general catalytic base in the acylation part of the reaction through deprotonation of Ser70. Ser70 then attacks the carbonyl carbon of the β-lactam ring. Glu166 is also thought to be involved in the second part de-acylation part of the reaction by activating a hydrolytic water molecule. Interestingly, the CTX-M-44 Glu166Ala (E166A) mutant is still able to degrade β-lactams, but with the rates several orders of magnitude slower [45]. This suggests there is more than one way to degrade β-lactams, including some that have been proposed to use Lys73 [46]. Neutron studies were conducted on the E166A mutant as it degrades the β-lactam slow enough to allow crystallographic investigation of the acyl-intermediate. Studies were done on both the ligand-free enzyme and in complex with cefotaxime. The data on the complex revealed that upon inhibitor binding, an oxyanion hole is formed involving the backbone amides of Ser237 and Ser70 with stabilizing H bonds to the carbonyl oxygen of cefotaxime. This interaction serves to stabilize the negative charge that is formed in the tetrahedral intermediates during the acylation and deacylation stages of hydrolysis.

**Figure 13:**
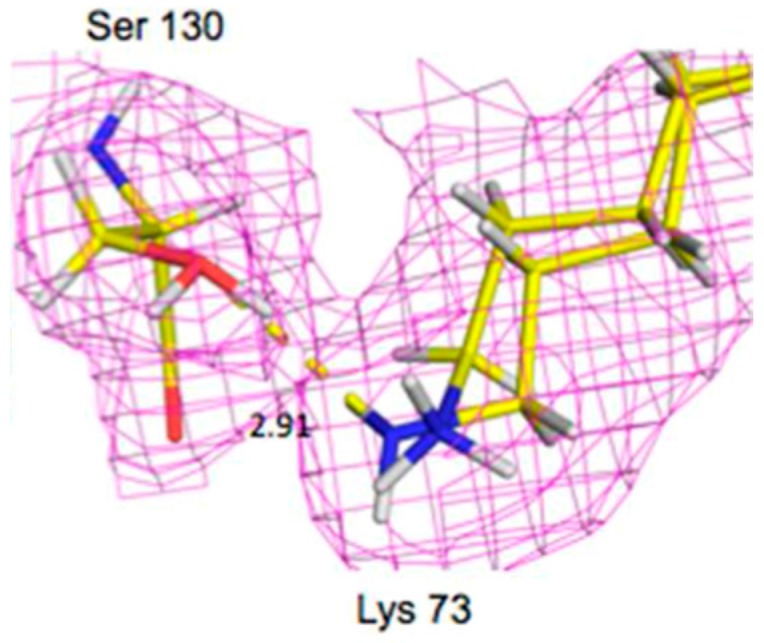
Proposed catalytic residues Lys73 in class A—lactamase could act as a general base. In the acyl-enzyme complex, Lys73 can be modeled in two conformations. The nuclear density map is shown in pink mesh and is contoured at 0.8σ. Reprinted (adapted) with permission from [45]. Copyright 2016 American Chemical Society.

Lys73 is highly conserved in class A β-lactamases and has been thought to act as general base, in competition with Glu166, by abstracting a proton from Ser70. The ability of E166A mutant to still produce the acyl-enzyme complexes, which supports the idea that Lys73 is involved, albeit less effectively than Glu166. In the ligand-free neutron structure, it is seen that Lys73 is charged (-D_3_^+^) and occupies a single conformation that is identical to that observed in the form where Glu166 is present. In the cefotaxime-complex structures however, Lys73 is observed in two alternative conformations that refine to near equal occupancy, presumably due to different charged states (Figure 13). These observations together support a role for Ly73 in proton transfer and represents a viable alternative pathway when Glu166 is mutated [45].

### 5.4. Diisopropyl Fluorophosphatase (DFPase)

The enzyme diisopropyl fluorophosphatase (DFPase; 314 residues, 35 kDa), found in the Mediterranean squid *Loligo vulgaris*, is a calcium-dependent enzyme that can hydrolyze a variety of toxic organophosphorus compounds through hydrolysis of a P-F bond. The compounds include the nerve agents Sarin, Soman, and cyclosarin, and the pesticide diisopropyl fluorophosphate (DFP). These agents act as irreversible inhibitors of acetylcholinesterase and serine proteases. The neutron structure of DFPase, together with a variety of high-resolution X-ray structures of wild-type and site-directed mutants, and enzymology have greatly clarified the mechanism [47,48].

**Figure 14:**
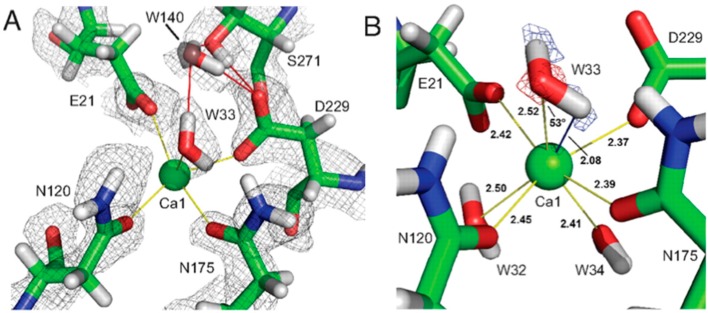
DFPase active site as observed in the neutron crystal structure. (**A**) 2F_o_ − F_c_ nuclear density (gray) shows a deprotonated Asp229; (**B**) Detail of the calcium-binding environment, showing the F_o_ − F_c_ omit density (gray) for the two D atoms in W33. This establishes the identity of W33 as water and not hydroxide. Details surrounding the active site calcium and associated water molecule enable mechanistic studies. Figure reproduced from [47].

Single- and multiple-turnover studies utilizing an ^18^O substrate, together with mass spectroscopy, showed a ^16^O-containing product under single-turnover conditions, and incorporation of ^18^O under multiple turnover conditions. After multiple turnovers, the enzyme was digested to determine where ^18^O had been incorporated and results showed that the proteolytic peptide in question contained the calcium-coordinating residue Asp229. These labeling experiments demonstrated that the reaction proceeded through a direct attack of an amino acid on the substrate, and not through a metal-activated water as previously thought. In the neutron structure, Asp229 was found to be deprotonated, consistent with the proposed mechanism (Figure 14A) [49].

The catalytic calcium ion is involved in 7-fold coordination, with a solvent molecule bound in the active site pocket. While X-ray structures were not able to discern the species of the solvent molecule, the neutron structure revealed it to be a water molecule, and not a hydroxyl, ascertained by the elongated nuclear density and F_o_ − F_c_ omit maps (Figure 14B) [47]. This unambiguously showed that the mechanism did not involve direct water activation by the calcium ion, as had been previously suggested by other investigators, but was consistent with the Asp229 in the role as nucleophile. Of note was the orientation of the bound water (D_2_O) molecule in relation to the position of the catalytic calcium ion, with a very close distance (2.08 Å) between one of the D atoms of the water and the calcium ion, and a Ca-O-D angle of 53 degrees.

### 5.5. Fungal Lytic Polysaccharide Monooxygenase

Fungal lytic polysaccharide monooxygenases (LPMO) are Cu-containing metalloenzymes involved with the degradation of polysaccharides through the insertion of one O into the glycosidic bond carbon, leading to chain cleavage. There is significant interest in the mechanism of these enzymes as there is potential application in cellulose degradation for biofuel production. The general mechanism starts with a single electron reducing the resting state Cu(II) to Cu(I). Cu(I) has a high affinity for molecular oxygen (O_2_). Oxygen binding to Cu(I) quickly regenerates Cu(II) through oxidation with the formation of Cu-superoxide. From this intermediate there are multiple possible pathways that may involve superoxide, hydroperoxyl, or oxyl that are responsible for abstracting an H atom from the glycosidic carbon. In the absence of substrate the superoxide species can leave Cu(II) and generate peroxide. There are many uncertainties in how these enzymes activate oxygen and the mechanism of peroxide production when no substrate is present [50,51].

*Neurospora crassa* LPMO is a 223 residue (23.3 kDa) enzyme with one Cu per monomer. High resolution X-ray crystallography and DFT calculations were supplemented with neutron crystallography in order to better understand mono-Cu activation of molecular oxygen and H_2_O_2_ production in the absence of substrate.

**Figure 15:**
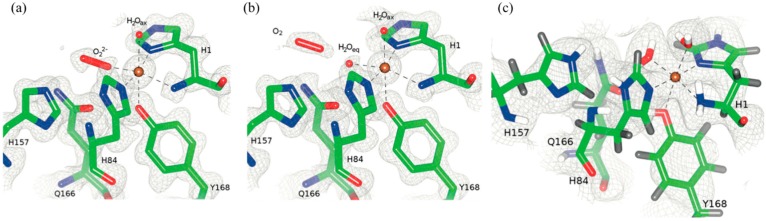
Cu-binding site and coordination in different crystal structures of LPMO from *Neurospora crassa*. (**a**) X-ray structure of crystal treated with ascorbate, peroxo (O_2_^2−^) bound; (**b**) X-ray structure of crystal treated with ascorbate, molecular oxygen (O_2_) bound in “pre-bound” position; (**c**) neutron structure with His157 modeled as neutral and pointing at “pre-bound” position. Figure reproduced with permission from [51].

X-ray crystal structures of the resting Cu(II) state showed that Cu is coordinated by a conserved “His brace” made of the backbone amide and imidazole of His1 and the imidazole of His84. The hydroxyl group of Tyr168 is pointing towards with Cu site with the remaining axial and equatorial coordination positions occupied by two waters, H_2_O_ax_ and H_2_O_eq_ (Figure 15) [51].

The coordination sphere for Cu indicates the presence of Cu(II), despite the possibility of photoreduction upon X-ray exposure. Treating the crystals with ascorbate in the presence of atmospheric oxygen prior to freezing leads to reduction to Cu(I) and formation of the Cu(II)-dioxo complex. The crystal structure shows that H_2_O_eq_ is displaced by a peroxo (O_2_^2−^) species in the equatorial position (bond length ~1.44 Å). This configuration is consistent with displacement by water to release peroxide in the absence of substrate. In the non-crystallographic symmetry related monomer molecular oxygen was instead observed, adjacent to H_2_O_ax_. The molecular oxygen in this position was proposed to be in a “pre-bound” position and not yet activated as was seen in the adjacent molecule [51].

The neutron crystal structure of the resting state showed a nearby His157 to be neutral. At lower pH this His157 would be protonation and positioned to coordinate molecular oxygen in this “pre-bound” location. This implies that His157 could possibly promote oxygen binding and activation. DFT calculations were performed on different His157 protonation states and conformations and showed a strong thermodynamically favored addition of oxygen when His157 is charged. Overall these studies revealed additional details about oxygen binding and activation not considered before and the observation of “pre-bound” molecular oxygen indicate a mechanism for H_2_O_2_ formation in the absence of substrate [51].

## 6. Ligand Binding and Substrate Specificity through H Bonds

Inhibitor binding to target proteins or enzymes can involve H atoms in different ways: through direct H bonding, water-mediated H bonding, C-H…π interactions, hydrophobic interactions, and electrostatic interactions (e.g., deprotonated Lys or His residues). In addition to the role of H atoms in the protein and solvent, the ligand’s own protonation state will have a large effect on solubility and binding properties. While X-ray crystallography has been a powerful tool in ligand binding studies and for rational drug design, it has limitations on the details of the information it can provide. Neutrons are severely underutilized in this field and can fill the knowledge gap by providing atomic information on ligand protonation and detailed information on how they bind to the target protein. 

### 6.1. Rational Drug Design Using Neutrons as a Guide

HIV protease (PR) is an aspartic protease and an integral part of the viral life cycle. PR cleaves the pre-cursor proteins Gag and Gag-Pol into functional, catalytic enzymes involved in viral replication and the structure of the viral capsid. Upon PR inhibition, the resulting virus particles are immature and non-infectious. As such PR is an important drug target for suppression and control of HIV infection and progression to AIDS. PR serves as a standout example of successful structure-based drug design efforts using X-ray crystallography. Since the first reported PR crystal structure, many PR inhibitors (PI) have been developed and are in use today [15]. Neutron studies of PR:PI complexes can give useful information to guide next generation structure-based drug design. Such analysis can reveal unfilled pockets in the active site, the role of water-mediated PI binding, and areas where H-bonding can be introduced, or removed, by chemical modification of new ligands.

PR is a homodimer with each monomer containing 99 amino acids. The PR active site is made up of a conserved triad, Asp25-Thr26-Gly27 and Asp25′-Thr26′-Gly27′ from the adjacent monomer. The two Asp residues from opposite monomers point at each other and substrate (peptides/proteins or inhibitors) binds between the catalytic Asp residues and the flexible flap regions. The first neutron structure of HIV PR in complex with a clinical drug, amprenavir (APV), was compared to another HIV PR neutron structure in complex with a non-clinical inhibitor KNI-272 [15,52]. In the APV complex neutron structure Asp25 is protonated and donates an H bond to the -OH group of APV. In contrast, Asp25′ accepts an H bond from the same -OH. It is interesting that overall the distribution of observed H bonds in the active site varies from the PR:KNI-272 structure. This shows that the location of H or D atoms on catalytic Asp residues, as well as the identity of H bond donors/acceptors, can be influenced strongly by the structure and chemistry of the PI.

Previous studies on ultra-high resolution X-ray structures enabled identification of a number of proposed H bonds between the ligand and PR [53]. These interactions are expected to enhance, or even enable, binding interactions. When the nuclear density maps for the PR:APV complex were studied, the inventory of actual H bonds were quite different from those inferred from the ultra-high resolution X-ray studies. In fact, some of the details seen in the neutron structure contradicted some of assigned H bonds and their relative importance in determining ligand binding. The neutron data shows only two strong H bonds. These are found between APV and catalytic Asp residues, and between the tetrahydrofuran moiety and backbone amides of Asp29 and Asp30. There is also a water-mediated H bond between Asp30′ and the flap regions. The result is three total H bonds and not four as was deduced from the X-ray data alone [15]. Previous ^13^C-NMR data showed that the catalytic Asp residues have mismatched p*K*_a_, and that this affects ionization and subsequent H bond potential. By direct investigation of the protonation states of Asp25 and Asp25′, the nuclear density maps support the NMR data in that only Asp25 is protonated [54]. Binding interactions can be improved by ligand derivatization to match the p*K*_a_’s. This can be achieved by incorporating a strong electron-accepting group in the APV molecule. Another strategy is to include chemical groups on the PI to directly engage PR in H bonds and minimize water-mediated H bonding. Such a strategy will also improve the enthalpy of binding.

In general the results from neutron studies highlight some issues with structure-based drug design. The overarching one is that X-ray data alone can be misleading from a rational drug design view. Neutrons can fill this gap by giving information on ionization/protonation state of residues, water, and H bonds involved. The data can also be used to estimate the importance of interactions based on distances and angles between donors/acceptors. Another issue is that the chemical and physical basis as to why some drugs bind better than others may be very subtle [15]. For these reasons it is important to use multiple methods or approaches to ligand/inhibitor binding interactions. Neutrons clearly have an important future role in structure-based drug design and this may be one of the best applications of the technique in structural biology. With new sources and improved instruments the practical feasibility of using NPX for drug design studies will only increase.

### 6.2. Ordered Water Network and H Bonds Provide a Template and Specificity for Substrate Binding

Carbohydrate binding modules (CBMs) are domains that are part of carbohydrate-metabolizing enzymes. They confer substrate specificity and increase enzymatic throughput by aiding enzymatic recognition of substrates and also by tethering the catalytic part of the enzyme close to the substrate [55]. Some CBMs only bind to a particular carbohydrate while others display more promiscuous binding and understanding the details will be useful in designing enzyme with specific properties, especially for industrial application (e.g., biorefinery processes). Neutron protein crystallography is a great tool for the investigation of substrate specificity. For example, it yields important clues in the case of one such engineered CBM, X-2 L110F, from a xylanase that is expressed in *Rhodothermus marinus* [56]. Wild type X-2 specifically binds to xylan. Mutation of a single residue Leu110 to a Phe (X-2 L110F) changes the substrate specificity to bind not only xylan, but also xyloglucan, and β-glucan. For the neutron studies the CBM was complexed to a branched heptasaccharide (XXXG) to probe the interactions to substrate that enable broader binding to more substrates. Even though H atoms are known to be important mediators of CBM binding to carbohydrates, there is little direct evidence of their involvement in H bonds, water mediated binding, π-stacking, and other electrostatic interactions. XXXG is a heptasaccharide that contains a chain of four glucose molecules and three branched xylose molecules.

Two of the glucose molecules in XXXG are involved in H bonds and a π-stacking interaction to two Phe residues while the other two are not engaged in any strong or visible interactions. An H bond was observed between Tyr149 and one of the branching xyloses, XYS1173, and explains why mutation of this residue disrupts binding ability to branched sugars. XYS1173 is also involved in two additional H bonds to His146 and Arg115 and in a π-stacking interaction with Pro68. Neutron data shows that Arg115 is oriented to donate the H bond through a network of interactions that results in optimal positioning for this interaction. The neutron studies revealed the interactions surrounding branched xylose XYS1173 and support the inability of the L110F mutant to bind sugars that are further substituted at this position (e.g., XLLG) [57].

High resolution X-ray crystallographic studies of wild type X-2 and apo X-2 L110F show a number of ordered water molecules engaged in a network occupy the ligand binding cleft [58]. At least ten of these waters are displaced upon ligand binding giving an entropic enhancement to binding. In addition, the oxygen atoms of these waters are precisely replaced by sugar oxygen atoms upon binding, suggesting that waters serve as a chemical template for ligand binding. Xylan and xyloglucan displace a similar number of waters, but XXXG removes an additional three water molecules and causes compensatory amino acid side chain movements. Three conserved water molecules that form an H bonded network were observed between BGC1171 and several ligand binding residues. Together these waters and the H bonds to the protein serve to orient the residues to be in optimal H bonding configurations.

This work highlighted several important considerations in carbohydrate binding interactions. Mainly it is proper positioning of interacting amino acid side chains and H bonds to ordered water that give clues as to how a carbohydrate ligand may bind. These factors are also involved in understanding substrate specificity and could be exploited to design CBM for specific carbohydrate recognition [57].

### 6.3. Water Displacement, Drug Ionization, and H Bonds in Mediating Drug Binding

Human carbonic anhydrases are expressed in diverse tissues and cell types throughout the body. Due to the fundamental nature of the catalyzed reaction, carbon dioxide hydration, CAs are involved in a number of physiological processes. They are at the center of many pH homeostasis processes, gluconeogenesis, bone remodeling, cerebrospinal fluid production and so on. One human isoform, HCA IX, has been found overexpressed in numerous cancer cells and are now an attractive drug target for cancer detection, imaging, staging, and treatment [59,60]. Carbonic anhydrase inhibitors (CAIs) have been prescribed for decades as diuretics and for the treatment of glaucoma. Today there are over twenty CAI in clinical use. In recent years their use has been expanded for use as anti-epilepsy and anti-obesity drugs. Clinically used CAIs have a common motif, a sulfonamide group that coordinates directly to the active site Zn^+2^, displacing a critical catalytic water molecule in the process [60,61]. HCAs share a high degree of sequence and structural identity, making it very challenging to design inhibitors that display isoform selectivity. In the case of HCA IX, it would be more clinically effective to have an inhibitor specific for HCA IX to minimize binding to the other HCAs with important, central physiological functions. Acetazolamide (AZM) is a classic CAI with a K_i_ of 12 nM for HCA II. AZM has three possible protonation states in solution. It was unknown which form binds to CA under physiological pH conditions. High-resolution X-ray crystallographic studies did not reveal the exact nature of the molecule in terms of ionization state or how specifically it binds to the active site of HCA II.

**Figure 16:**
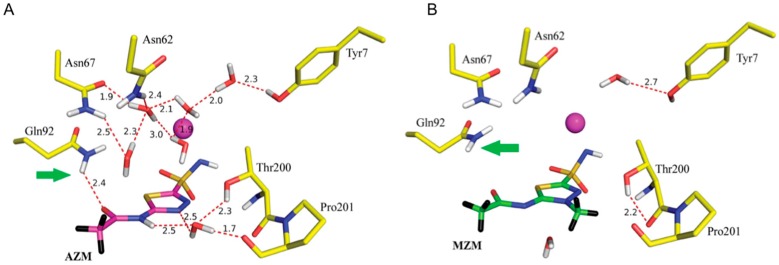
Comparison of AZM and MZM binding and water displacement as observed in neutron crystallographic studies. (**A**) AZM complex; (**B**) MZM complex [60]. Figure reproduced, with permission from the International Union of Crystallography from [60].

A 2.0 Å neutron structure of HCA II in complex with AZM revealed that AZM in this complex is anionic with the unprotonated sulfonamide group coordinated to the active site zinc. In contrast to the negatively charged sulfonamide group, the acetamido group is protonated with clearly visible nuclear density for the exchanged D atom at this position (Figure 16A). The single D atom on the sulfonamide group is engaged as H bond donor to Thr199. Thr200 acts as a bifurcated H bond donor to an ordered solvent molecule and the backbone of Pro201. In this way the solvent molecule is poised to act as H bond acceptor from the acetamido group of AZM. A total of four water molecules are displaced upon AZM binding and this will lead to entropic gains in drug binding. Finally the data also showed two C-H…π interactions. The first is between the AZM thiadiazole ring and Leu198, the other between the AZM methyl group and Pro201 itself [59,60].

Another neutron study investigated the drug methazolamide (MZM) bound to HCA II [60]. MZM is a methyl derivative of AZM and has superior properties in terms of shelf life, stability, and is less water-soluble than AZM, making it better suited for use in glaucoma treatment. Unsurprisingly the data showed that MZM was also unprotonated on the sulfonamide moiety and has similar coordination to the zinc as AZM (Figure 16B). MZM displaced a total of seven water molecules, compared to four by AZM. Three of the water molecules are common between the two structures. This means that in the AZM complex there are still a number of ordered, H bonded water molecules that are absent in the room temperature MZM complex. In the MZM situation there is an enthalpic trade-off that involves the breaking of so many H bonds to expel the waters, this balances the entropic gain of releasing water to the bulk solvent. This explains why the K_i_ for HCA II of MZM is still very similar as AZM (i.e., ~10 nM). The added methyl group on the thiadiazole group of MZM disrupts the water-mediated H bond that was observed between AZM and Thr200 and Pro201 [59,60]. Detailed knowledge of H bonds and water networks in ligand binding is valuable and neutron crystallography will have increasing importance in rational drug design that can exploit this knowledge. Having quantitative information on H bonds can be used for example to modify or derivatize compounds to introduce compensatory interactions or to produce a desired ionization form of the ligand.

## 7. Conclusions

This review highlights specific areas of structural enzymology and general protein biochemistry where neutrons have made a large impact. Due to their unique nature and interactions with biological materials, neutrons have the ability to give high resolution, detailed, unambiguous information on H atoms and their all-important interaction, the H bond. Neutron crystallography has provided the opportunity to “see” into the heart of the enzyme active sites or ligand binding regions, and often brings new insights. Neutrons have contributed direct evidence of amino acid involvement in catalysis or drug binding, how waters are organized and oriented, and even how some waters are activated to perform catalysis. Arguably the most important thing neutrons can show us is the H bond and how it ties the protein, solvent, and/or ligand together. As illustrated in this review, neutron crystallography complements results from NMR spectroscopy, X-ray crystallography, modeling, and computational chemistry. In recent years, significant advances have been made in the fields of DFT calculations and ^1^H-NMR spectroscopy for the study of H bonds [62,63]. It can be expected that theoretical and experimental information gained from all these methods will impact how protein structures are refined and analyzed.

Neutron crystallography is under-utilized by the general structural biology community due to perceived issues with sample preparation and acquiring beam time. With current molecular biology tools and equipment for protein production, purification and crystallization, there are now far fewer technical limitations to preparing sufficient material to grow large enough crystals for neutron diffraction experiments. Current instruments routinely collect high quality data from crystals that are 0.1–1 mm^3^ and smaller in volume, a range that is readily attainable for many systems. The low number of instruments and highly competitive nature of being awarded beam time, especially compared to macromolecular X-ray beamlines, represents the true bottleneck for current and new users. With the advent of the new European Spallation Source (Lund, Sweden), and the planned Macromolecular Diffractometer NMX there, data collection and sample requirements will dramatically improve for the user community.

## Figures and Tables

**Table 1:** Neutron and X-ray scattering lengths for atom types commonly found in proteins **^§^**.

Isotope	Z	Neutron Scattering Length (10^−13^ cm)	Incoherent Cross Section σ_i_ (10^−28^ m^2^)	X-ray Scattering Length (10^−13^ cm), sin θ = 0	X-ray Scattering Length (10^−13^ cm), (sin θ)/λ = 0.5 Å^−1^
^1^H	1	−3.74	80.27	2.80	0.20
^2^H (D)	1	6.67	2.05	2.80	0.20
^12^C	6	6.65	0.00	16.90	4.80
^14^N	7	9.37	0.50	19.70	5.30
^16^O	8	5.80	0.00	22.50	6.20
^32^S	16	2.80	0.00	45.00	1.90

**^§^** Taken from [11,13].
